# Supplementary material for: Enterovirus particles expel capsid pentamers to enable genome release
Source: Nat Commun. 2019 Mar 8;10:1138. doi: 10.1038/s41467-019-09132-x (PMC6408523; doi:10.1038/s41467-019-09132-x)
Supplement: Supplementary file 1 — Supplementary Information [file 41467_2019_9132_MOESM1_ESM.pdf]

Supplementary information for:

Enterovirus particles expel capsid pentamers to enable genome release

David Buchta[1], Tibor Füzik[1], Dominik Hrebík[1], Yevgen Levdansky[1,3], Lukáš Sukeník[1,2], Liya Mukhamedova[1], Jana Moravcová[1], Robert Vácha[1,2], Pavel Plevka[1]

[1] Central European Institute of Technology, Masaryk University, Brno, Czech Republic
[2] Faculty of Science, Masaryk University, Brno, Czech Republic
[3] Current address: Max Planck Institute for Developmental Biology, Tübingen, Germany

Correspondence and requests for materials should be addressed to P.P. (email: pavel.plevka@ceitec.muni.cz)

Supplementary figures:

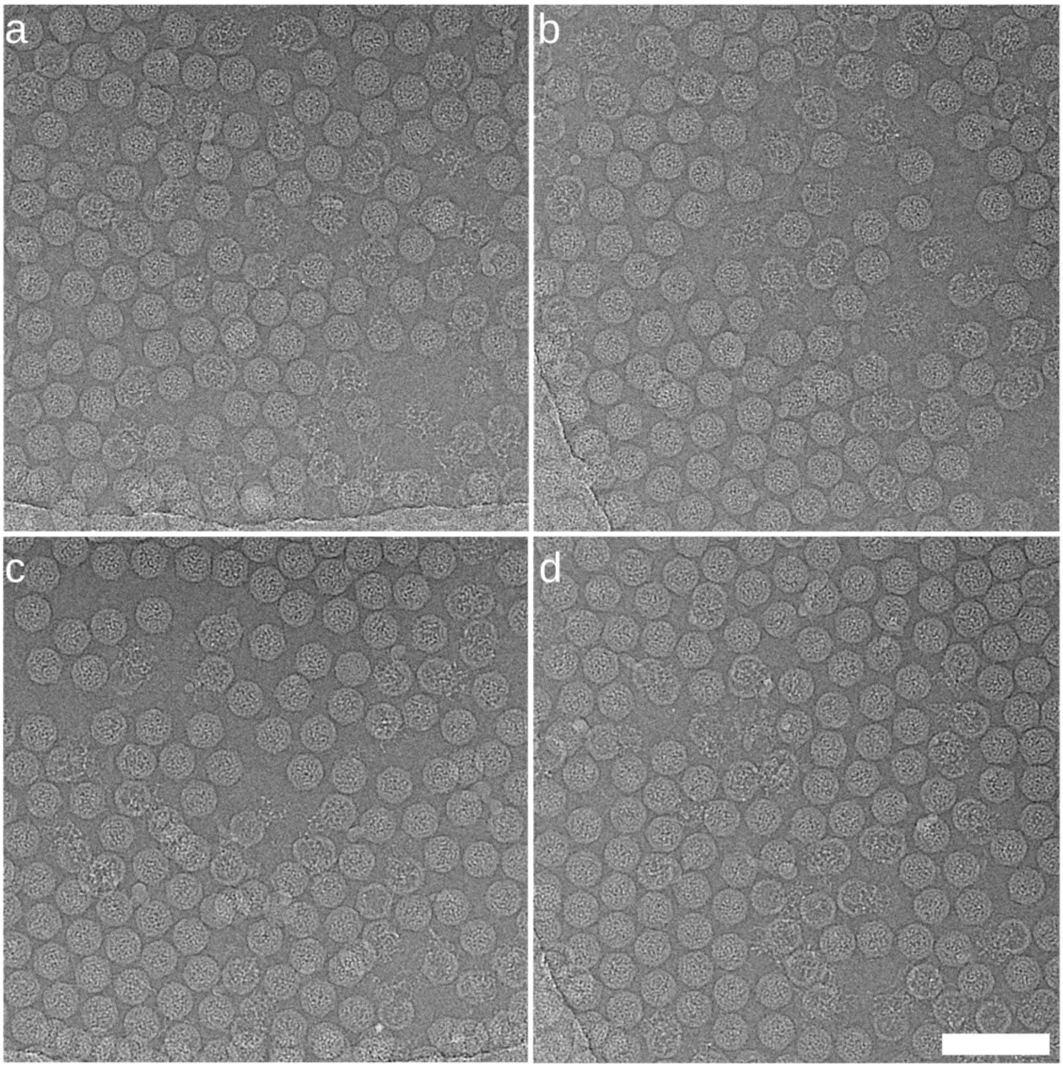

Supplementary figure 1. Cryo-electron micrographs of echovirus 18 particles captured in the process of genome release after incubation at acidic pH. The four images (a-d) demonstrate that the distribution of the genome release intermediates is random with respect to the centers of quantifoil holes. Therefore, the putative gradient of vitreous ice in the grid hole has no impact on the genome release of enterovirus 18. Scale bar represents 100 nm.

Supplementary figure 2. Diagrams of single particle data analyses. Scheme of image processing, classification and reconstruction of cryo-EM images of particles of echovirus 18 (a) and echovirus 30 (b).

a

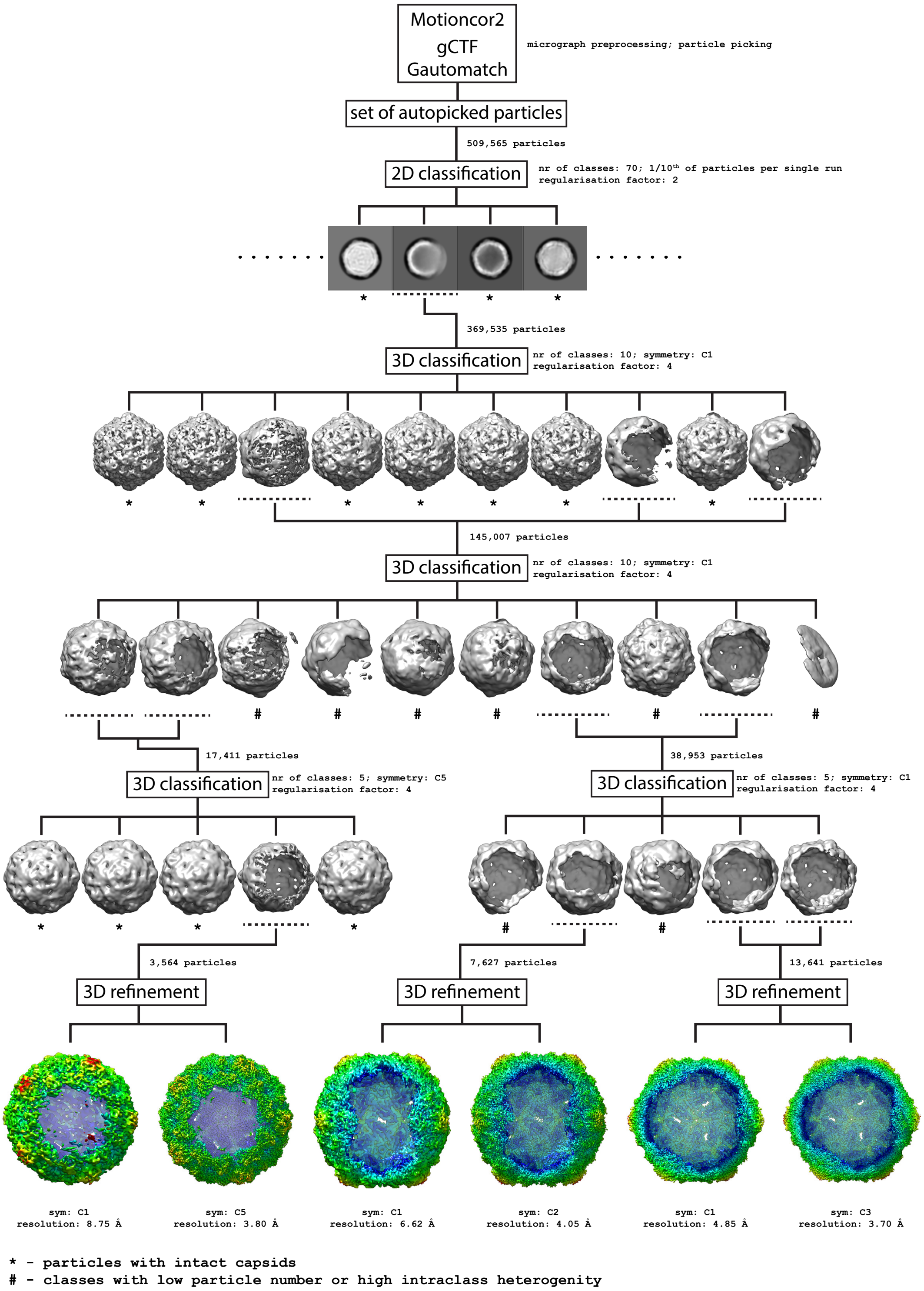

Supplementary figure 2. Continued

b

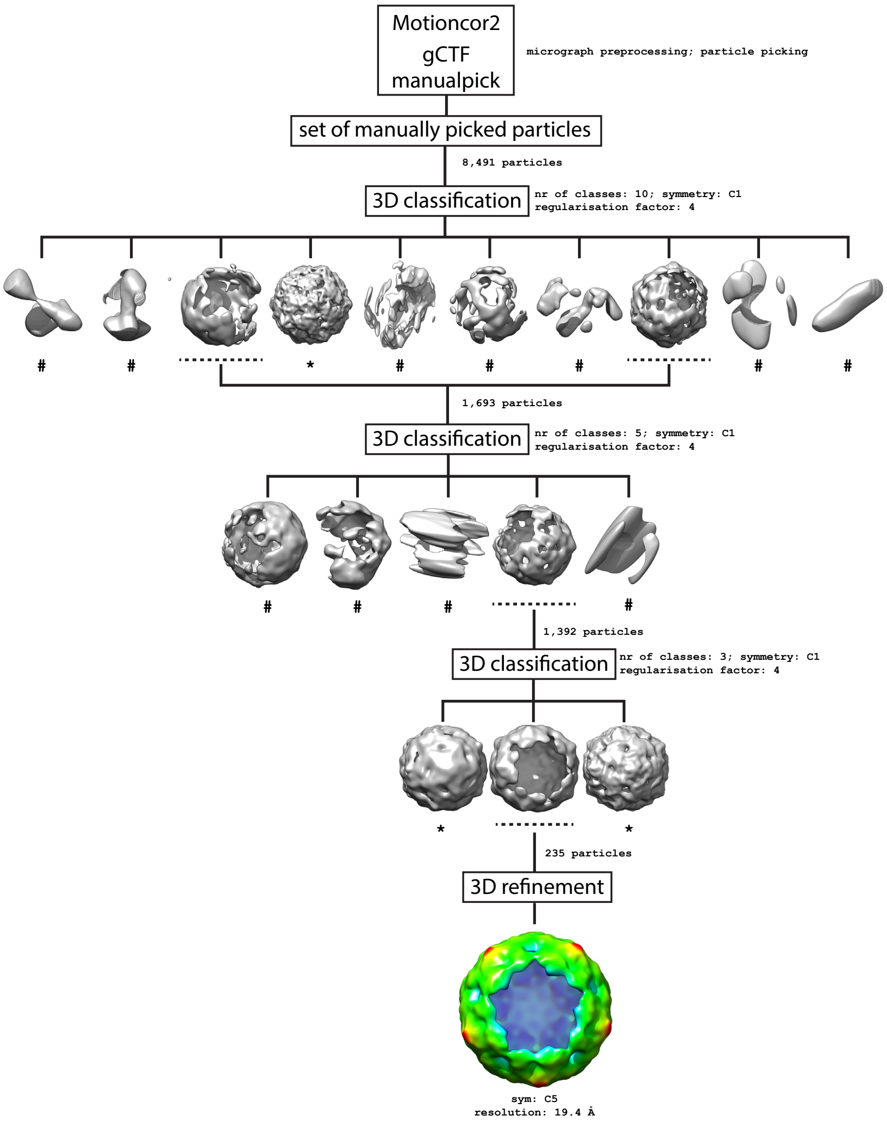

* - particles with intact capsids
\# - classes with low particle number or high intraclass heterogenity

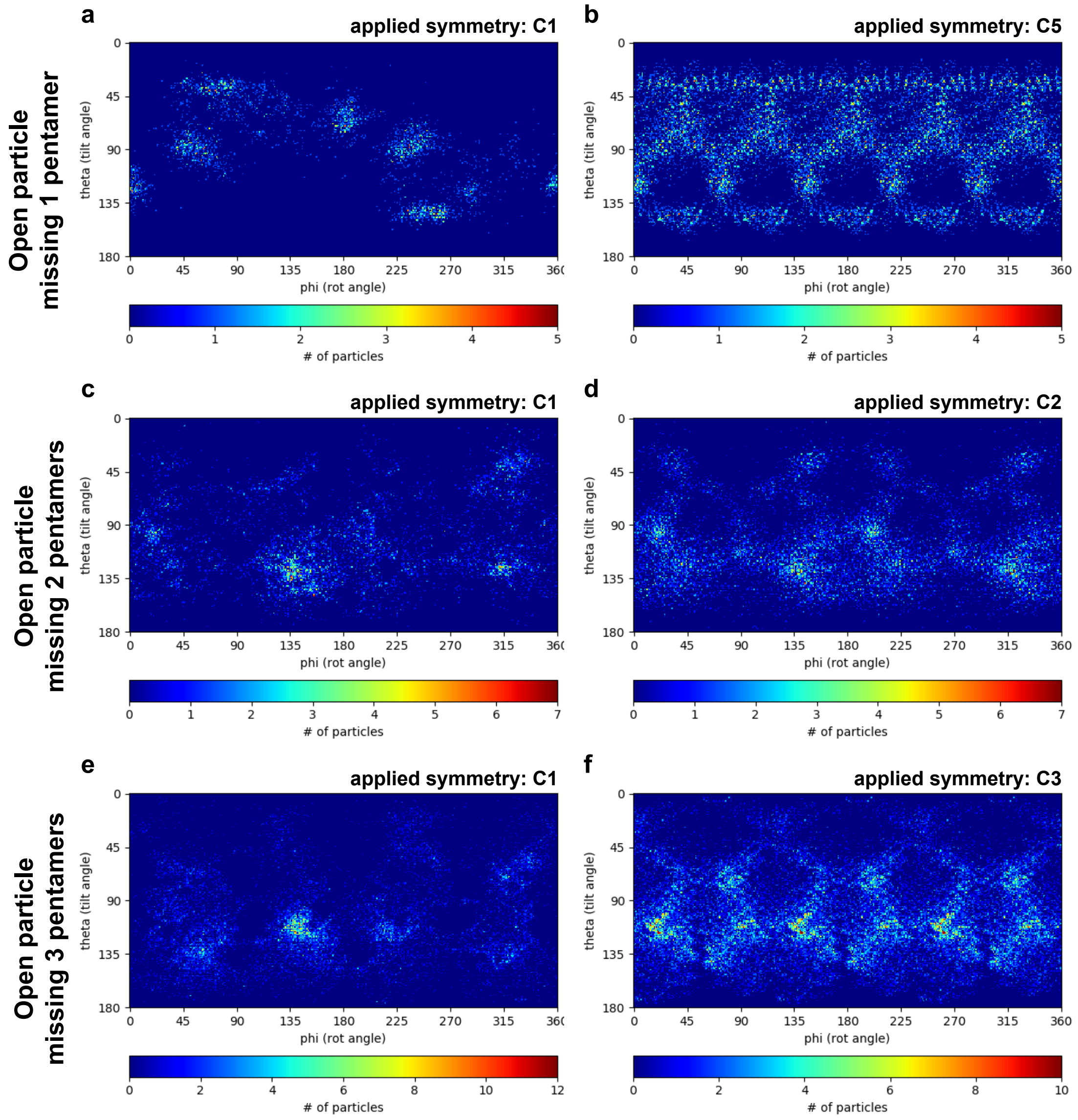

Supplementary figure 3. Angular distributions in reconstructions of echovirus 18 open particle. Heat-maps of angular distributions of particles in the final rounds of reconstructions of echovirus 18 open particles. Angular distributions of particles used in asymmetric reconstructions are on the left and those used in symmetrized reconstructions on the right.

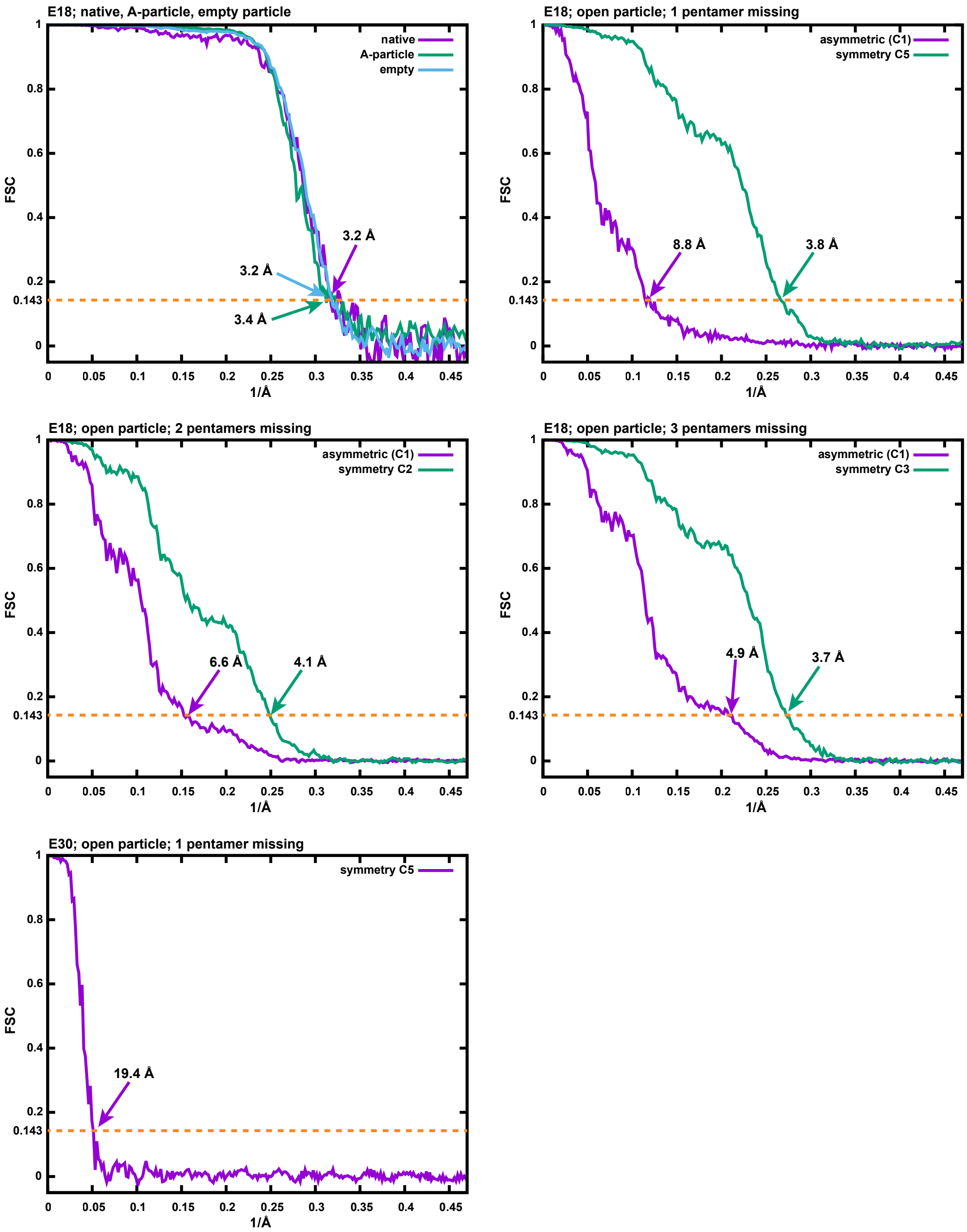

Supplementary figure 4. FSC curves of cryo-EM reconstructions of echovirus 18 and echovirus 30.

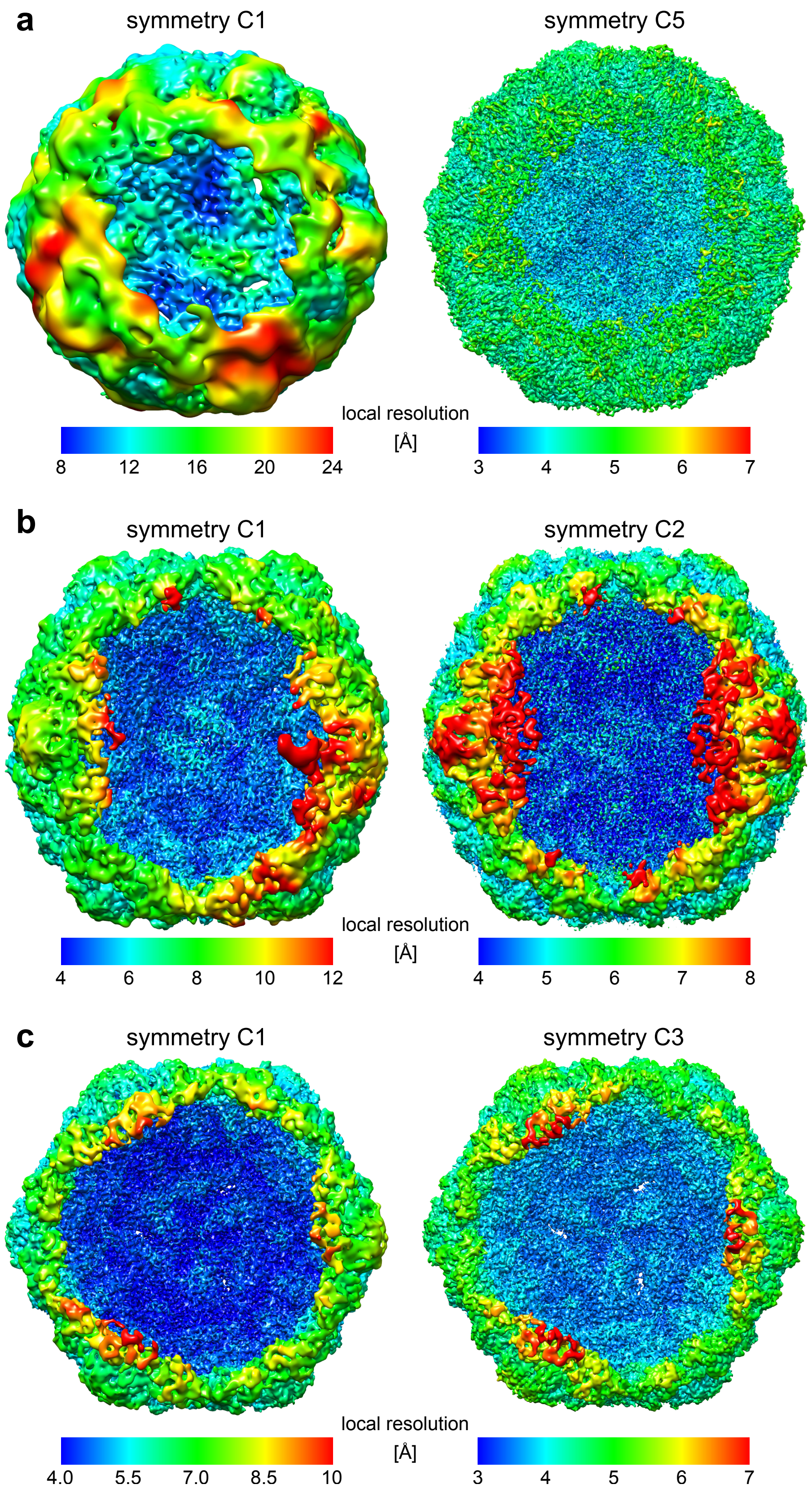

Supplementary figure 5. Local resolution maps. Local resolution maps of asymmetric (left column) and symmetrized (right column) reconstructions of echovirus 18 open particles lacking one (a), two (b), or three (c) pentamers.

Supplementary figure 6. Distribution of charged areas at inter-pentamer contacts of picornaviruses with known structures. Molecular surface representations of subunits at the contacts between two pentamers in native virion of echovirus 18 viewed from the outside (a) and inside (b) of the particle. Subunits from two icosahedral asymmetric units of each pentamer that form contacts across the inter-pentamer interface are shown in bright colors in blue for VP1, green for VP2, red for VP3, and yellow for VP4. The edges of the pentamers are highlighted with black lines. The plots of charge distributions of native virions at inter-pentamer contacts viewed from the outside (c) and inside (d). The plots of hydrophobicity distributions of native virions viewed from the outside (e) and inside (f). The plots of charge and hydrophobicity distributions of activated particles at inter-pentamer contacts (g). Panels (c-g) show only the subunits displayed in bright colors in (a,b).

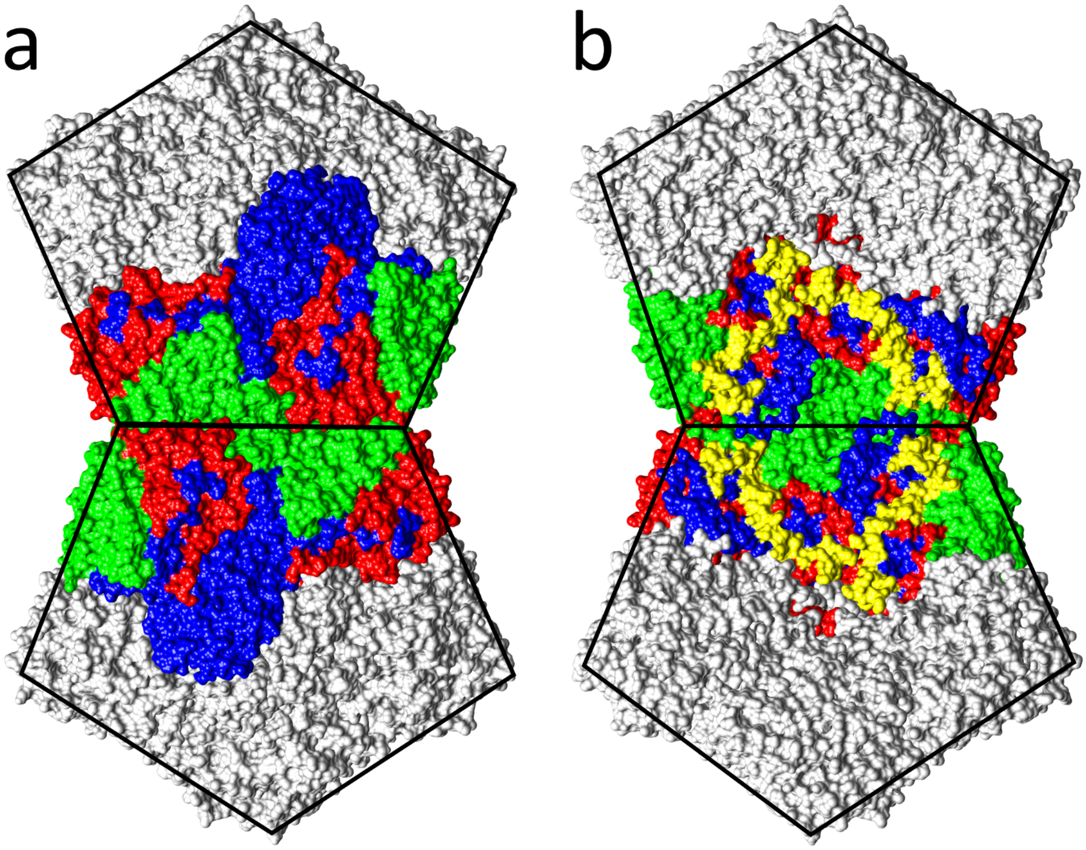

Supplementary figure 6c

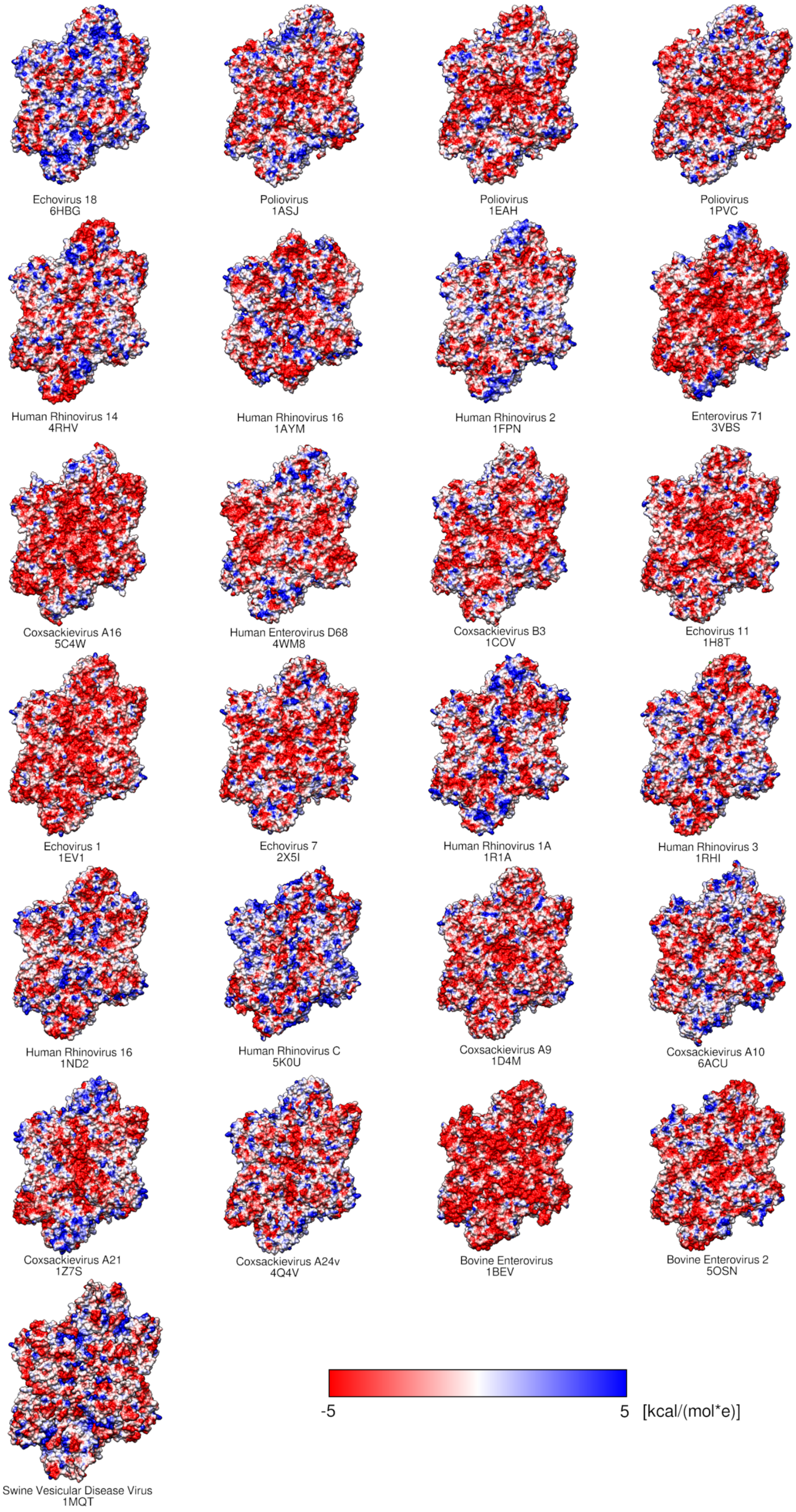

Supplementary figure 6d

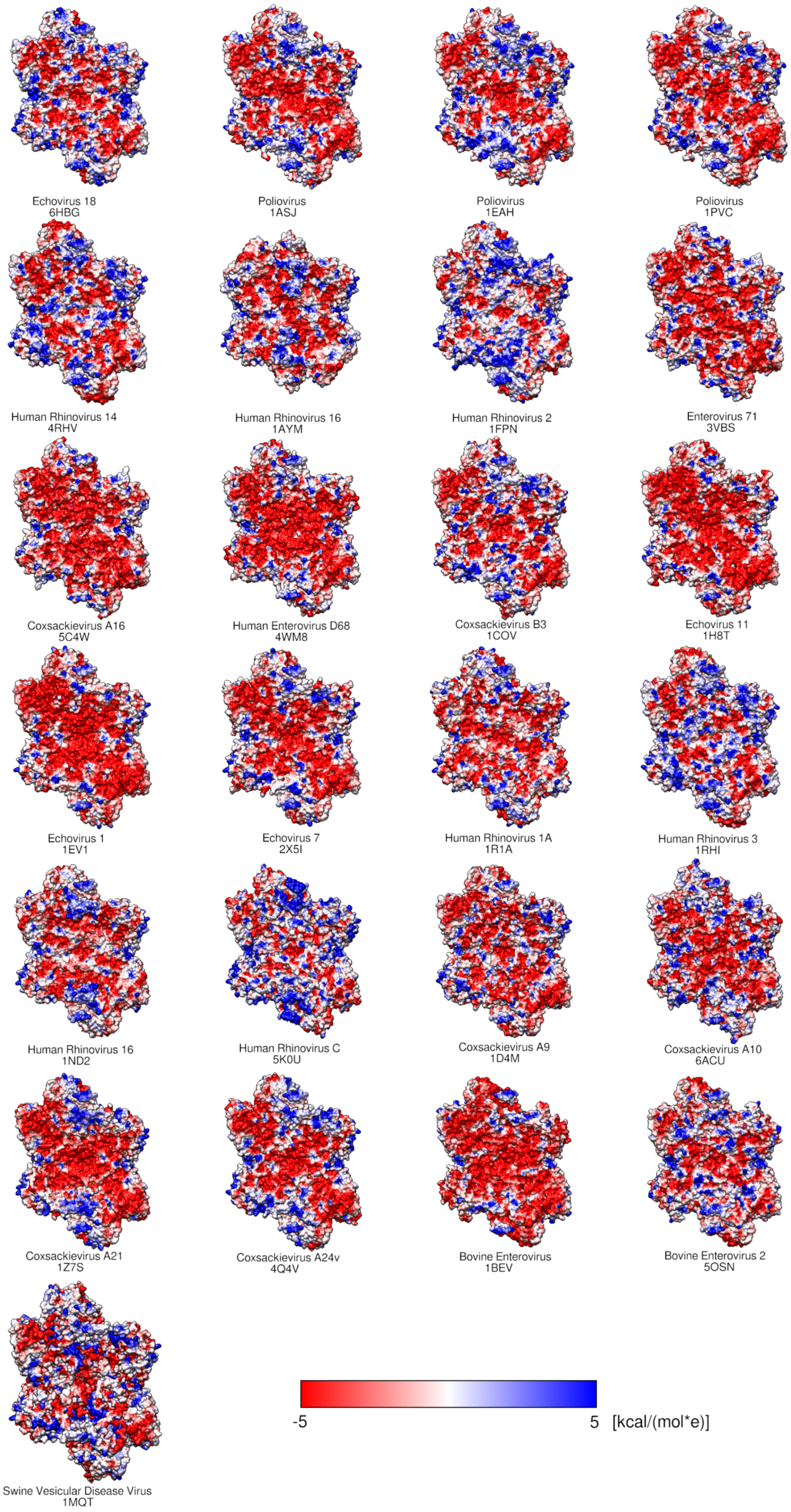

Supplementary figure 6e

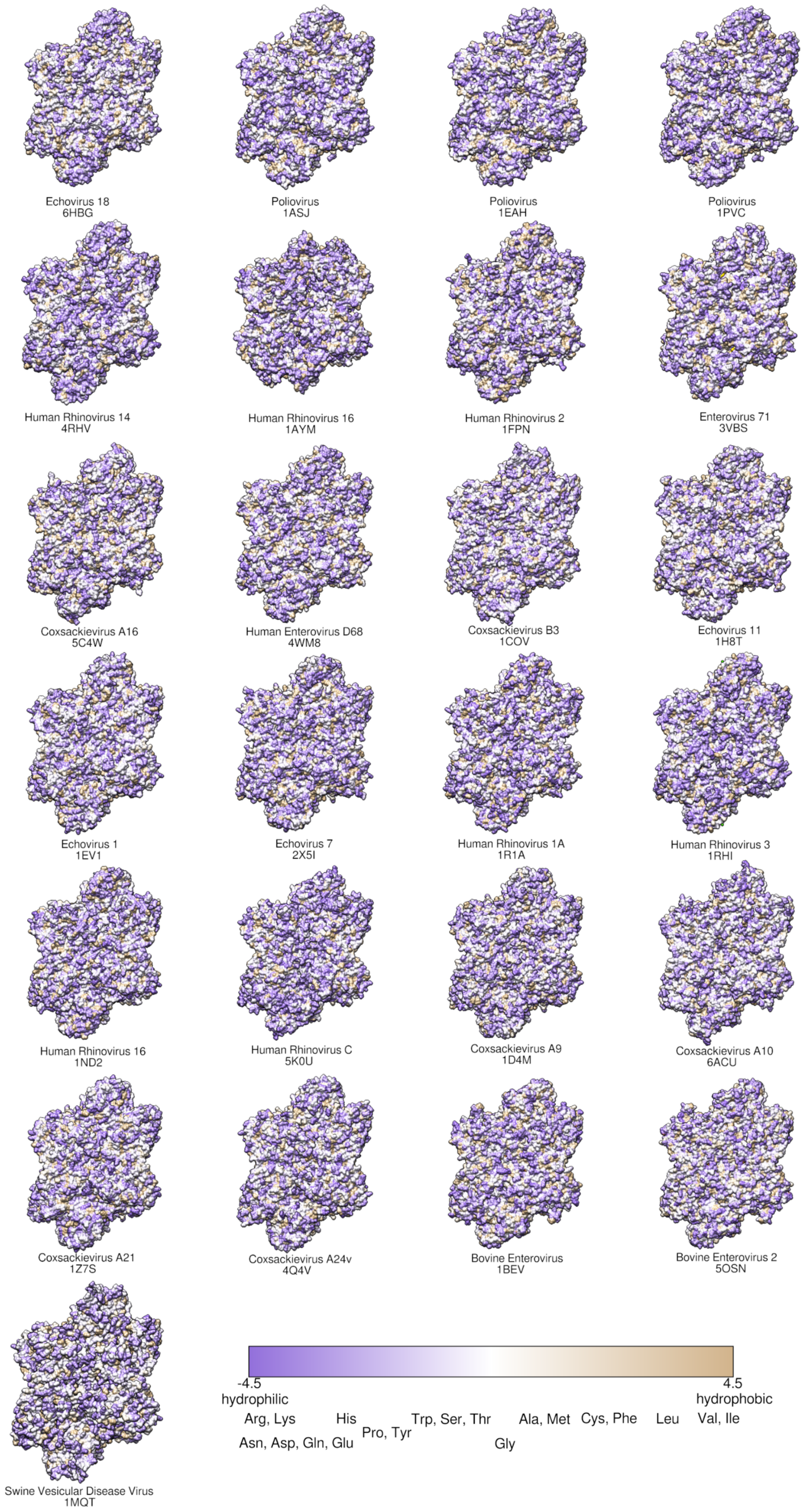

Supplementary figure 6f

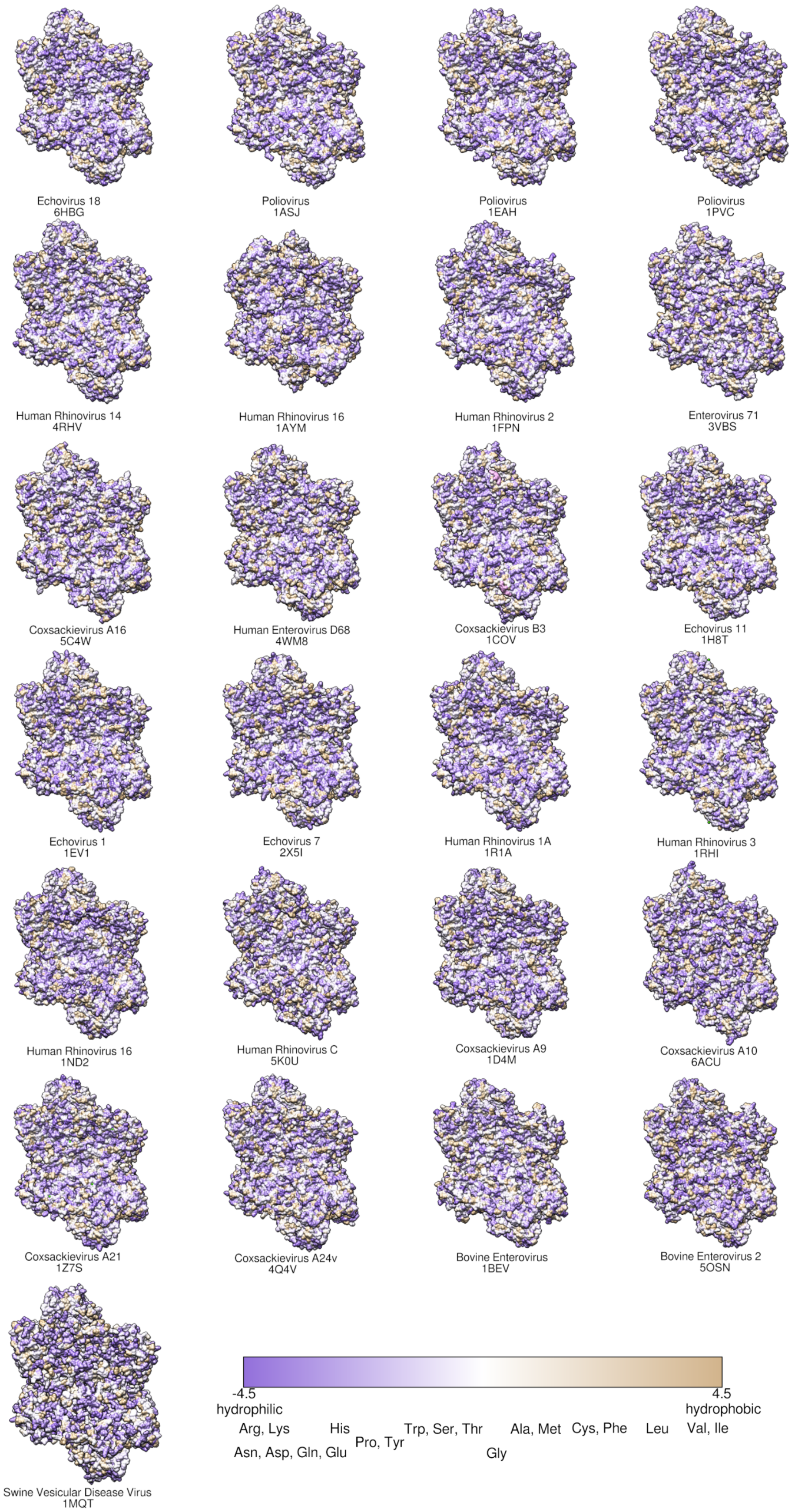

Supplementary figure 6g

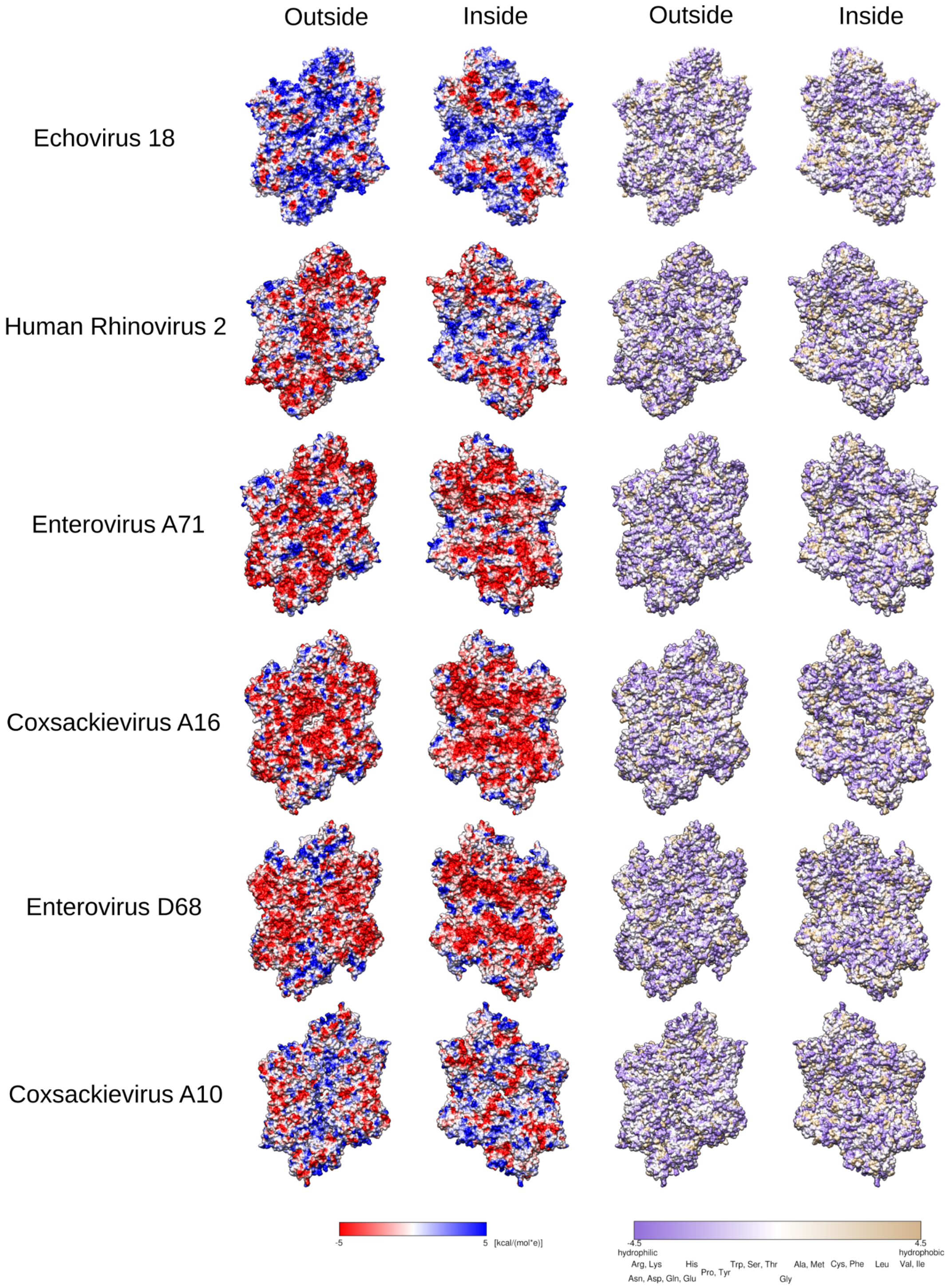

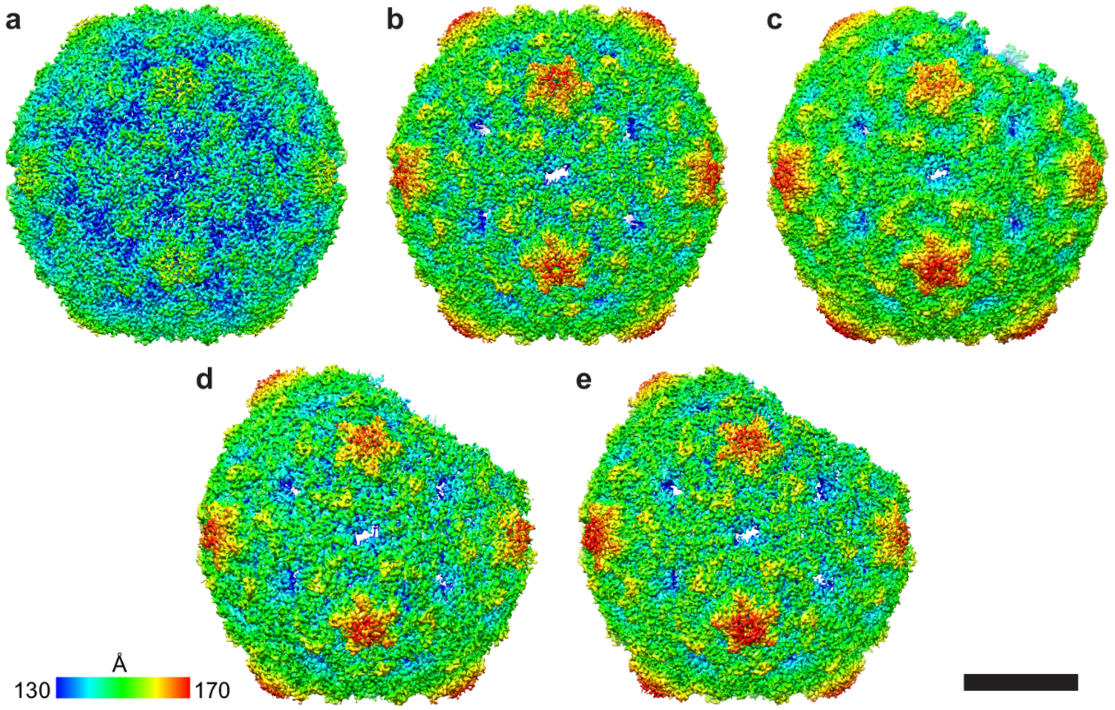

Supplementary figure 7. Comparison of of native virions and genome release intermediates of echovirus 18. Cryo-EM electron density maps of echovirus 18 native virion (a), activated particle (b), and open particles missing one (c), two (d), or three (e) pentamers (calculated with applied fivefold, twofold, and threefold symmetries, respectively). The surfaces of the particles are rainbow-colored based on the distance of the surface from the particle center. Scale bar represents 10 nm.

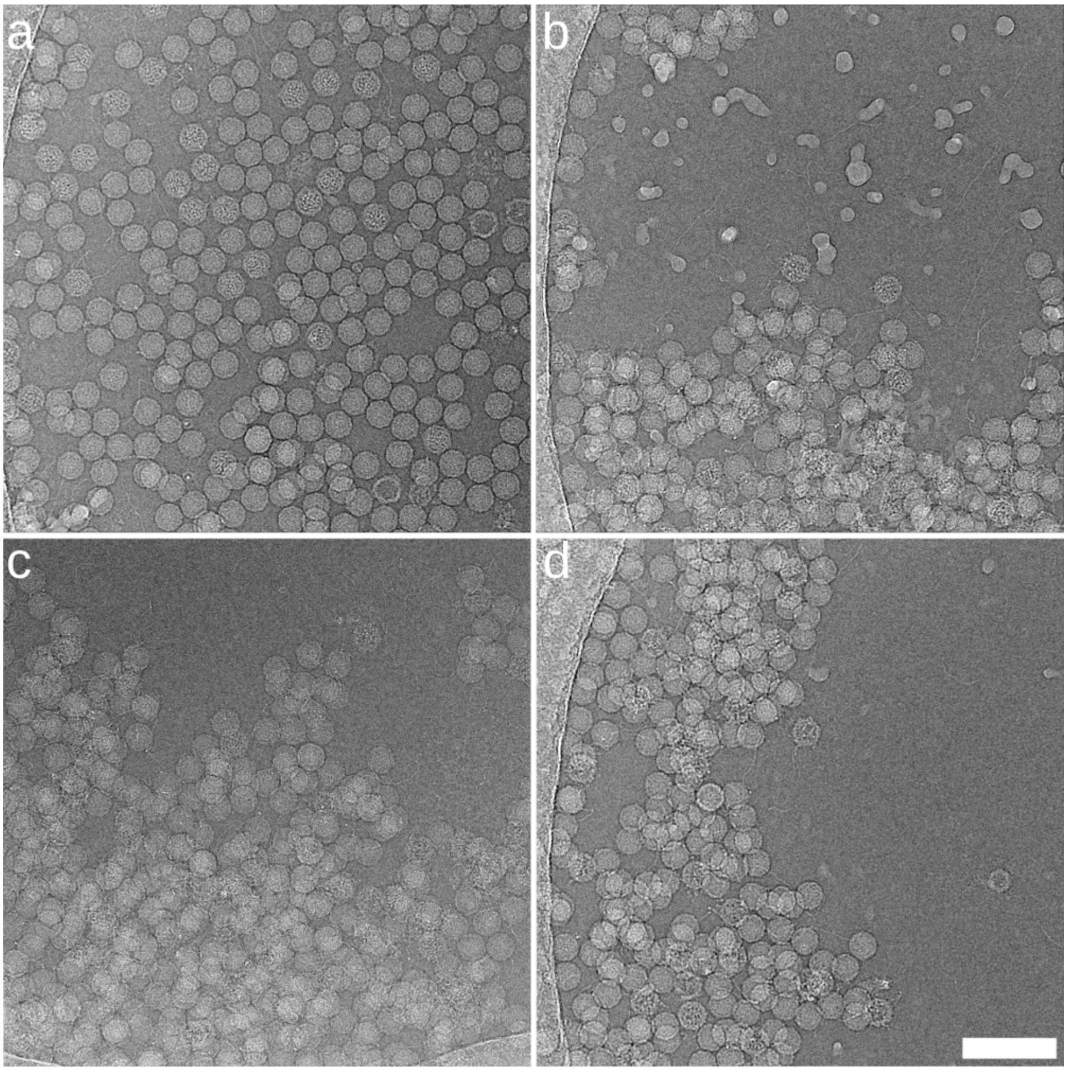

Supplementary figure 8. Exposure of virions of echovirus 18 to pH 6.0 at 37°C induces formation of activated particles and genome release within three minutes. (a) Native virions of echovirus 18 at neutral pH (control). Particles of echovirus 18 exposed to pH 6.0 at 37°C for 3 minutes (b), 12 minutes (c), and 21 minutes (d). Scale bar represents 100 nm.

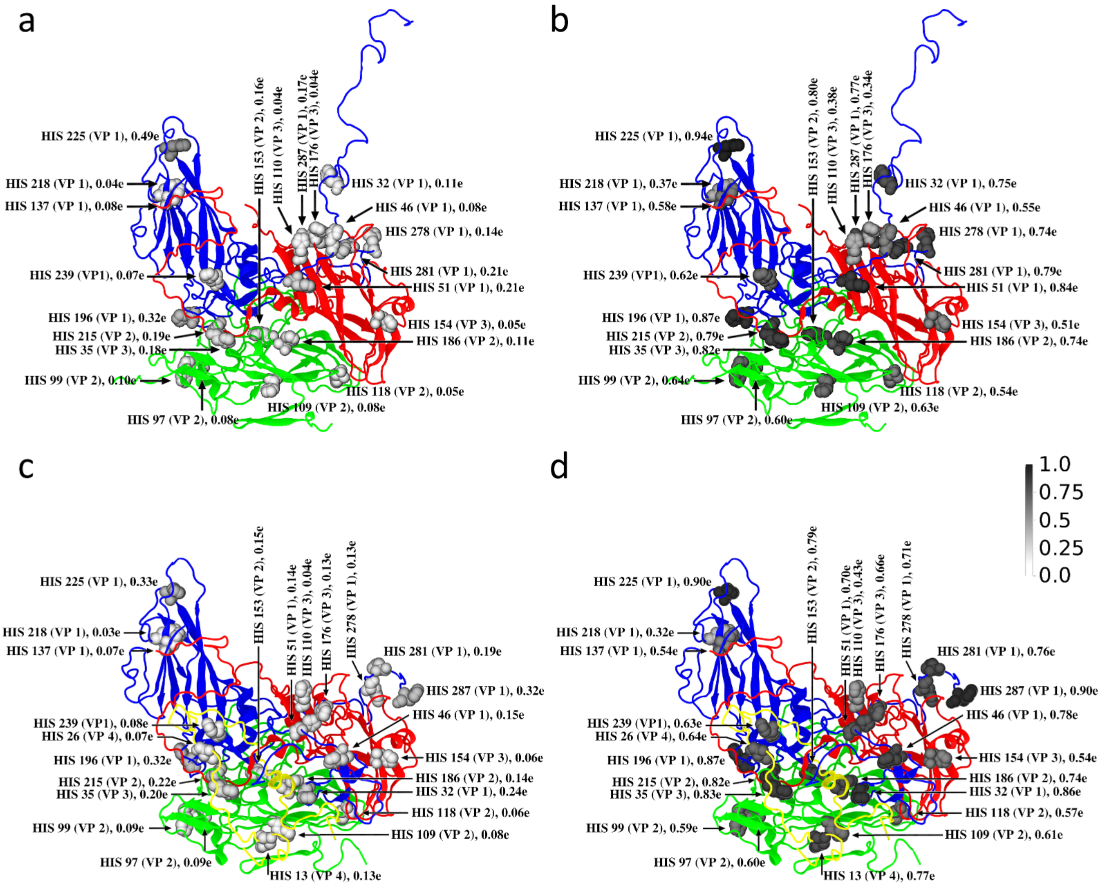

Supplementary figure 9. Impact of pH on changes of protonation of histidines in echovirus 18 capsid. Differences in protonation of histidines of capsid proteins of echovirus 18 virion (a,b) and activated particle (c,d) at pH 7.4 (a,c) and 6.0 (b,d). Partial charges of histidines are indicated by grey-scale and listed in description.

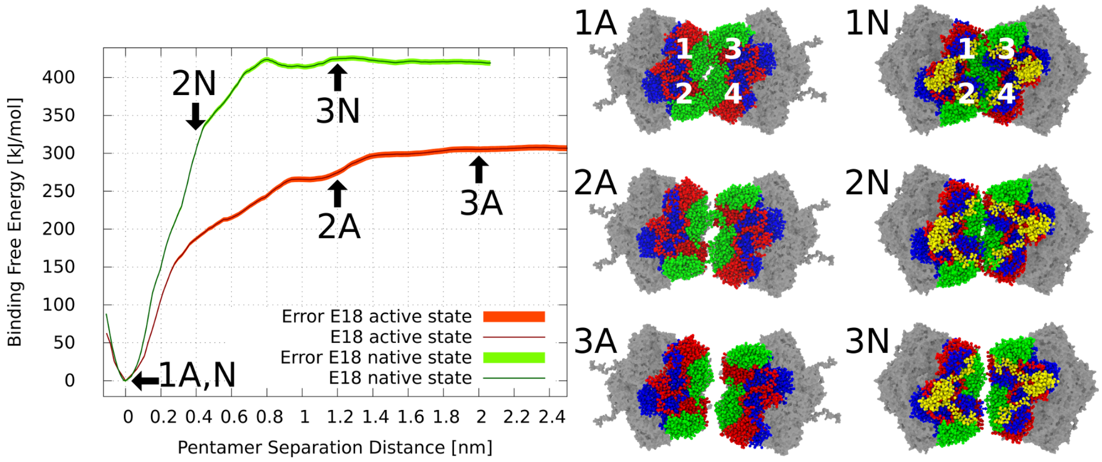

Supplementary figure 10. Binding free energy of two pentamers across twofold axis. The binding free energy is higher and more short-ranged in the native virion of echovirus 18 than in the activated particle. In step 1), both the activated particle (A) and native virion (N) states have minimal free energy which corresponds to the structures obtained using cryo-EM. The interaction is more long-ranged for the activated particle due to the interactions of flexible N-terminal chains, as shown in step (2A). In contrast, the pentamers of the native virion at the same separation are already too far apart to interact (3N).

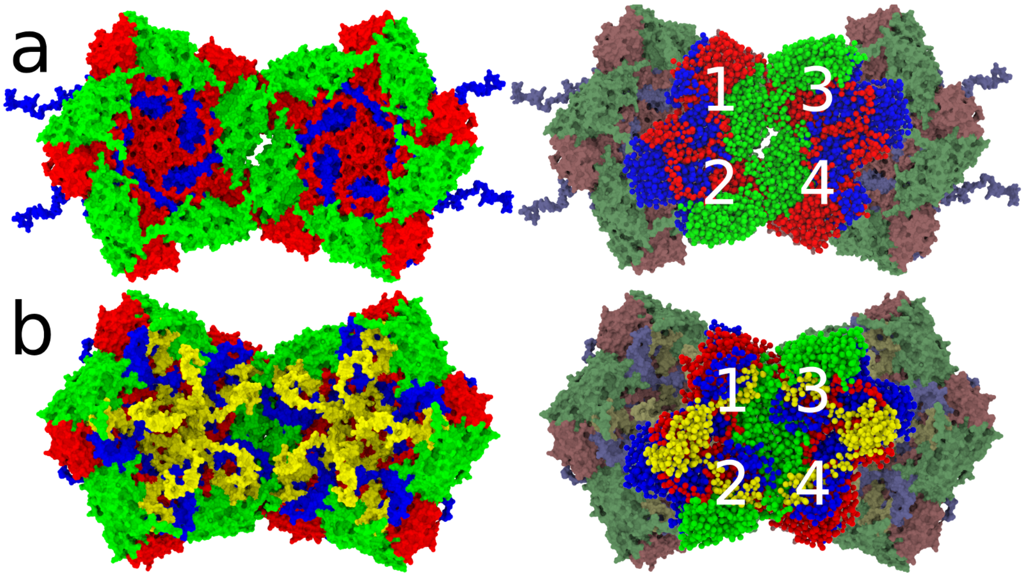

Supplementary figure 11. Interactions of pentamers of capsid protein protomers of human echovirus 18. Activated particle (a) and native virion (b). The left column consists of an all-atom representation. On the right side the protomers used for the interaction energy calculation in the Martini force field are highlighted. Color-coding: VP1 is blue, VP2 is green, VP3 is red, and VP4 is yellow.

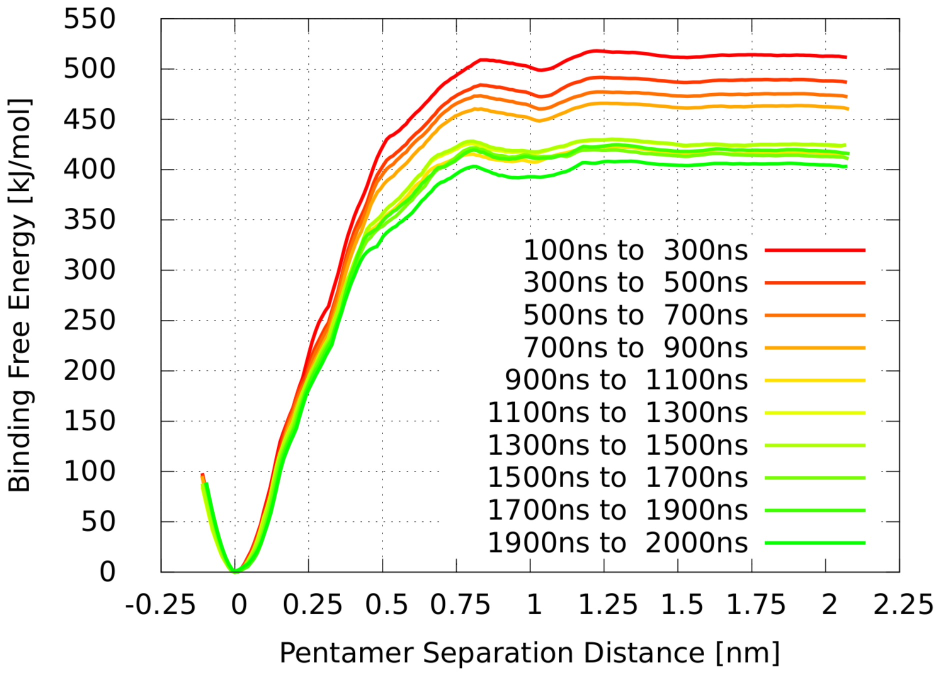

Supplementary figure 12. Binding free energy profiles of two pentamers. More than 1500 ns were necessary for each umbrella window simulation.

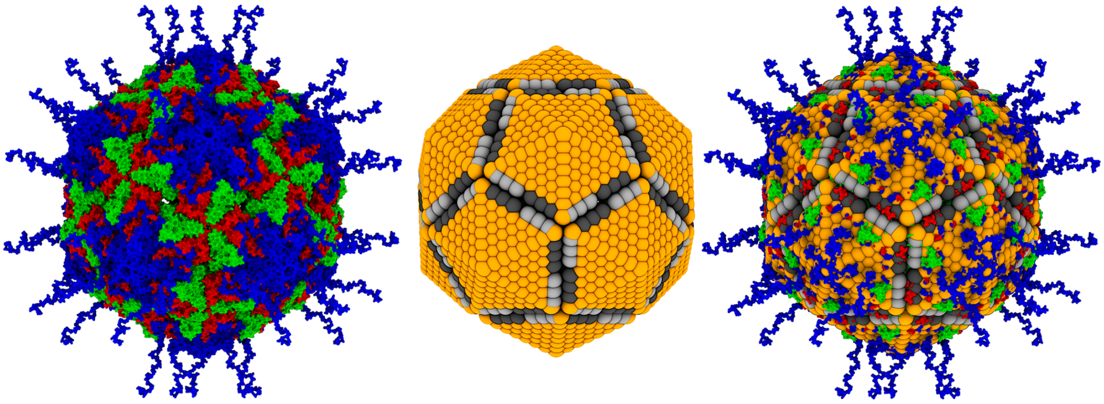

Supplementary figure 13. Coarse-grained model of echovirus 18 particle. Images of activated particle of echovirus 18 (left) and coarse-grained model (middle). The right column is an overlay of both structures. Color-coding: VP1 is blue, VP2 is green, and VP3 is red. The coarse-grained model was made of purely repulsive bead types (orange), forming the body of the pentamer subunit. Interactions between the pentamers were mediated by weakly attractive gray beads and strongly attractive red and green beads.

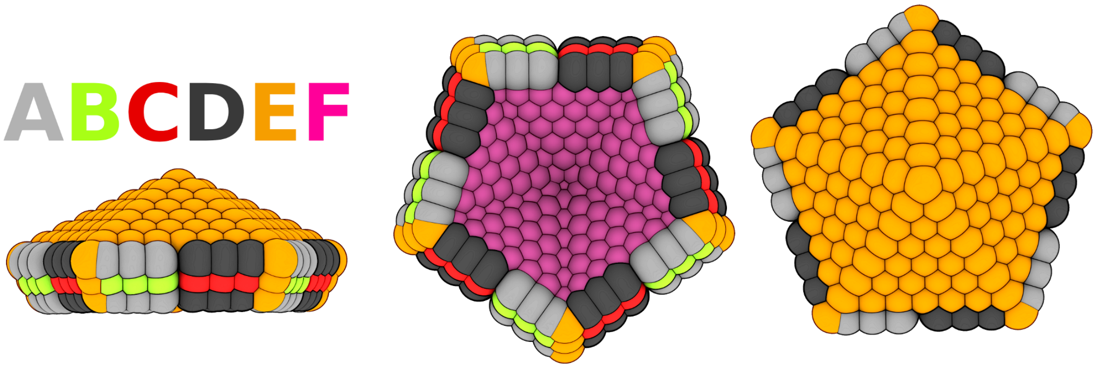

Supplementary figure 14. Phenomenological model of pentamers of human echovirus 18. The model is displayed from side (left), inner (middle), and outer (right) view. Coarse-grained model was made of purely repulsive bead types, orange (E) and purple (F), forming the outer and inner body of the pentamer subunit, respectively. Interactions between the pentamers were via weakly attractive gray beads (A and D) and strongly attractive red and green beads (B and C types).

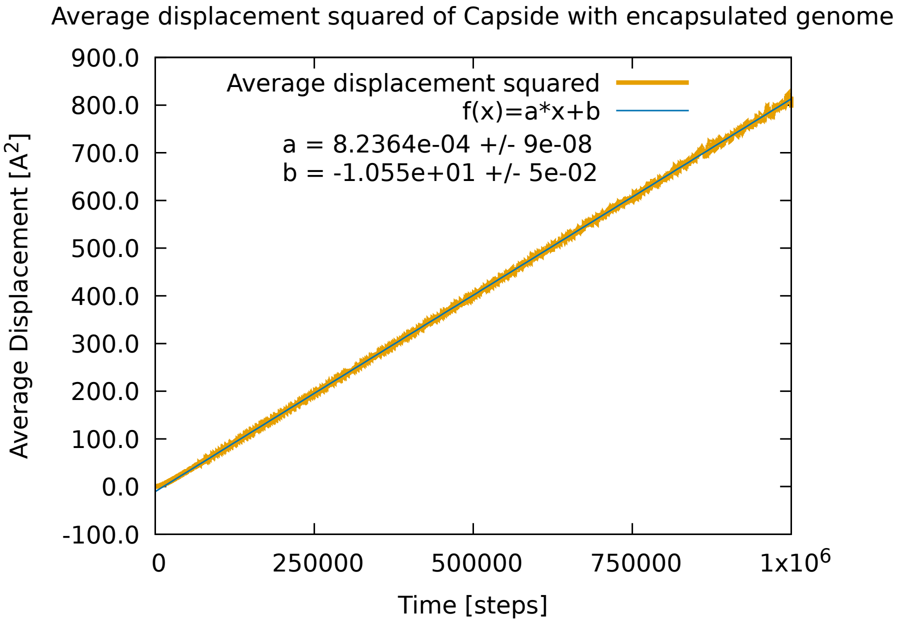

Supplementary figure 15. Average displacement of full capsid center of mass as a function of simulation time. A linear fit was used to determine the diffusion coefficient.

Supplementary tables

Supplementary table 1. Cryo-EM structure quality indicators.

	Echovirus 18 native virion	Echovirus 18 activated particle	Echovirus 18 empty particle	Echovirus 18 missing one pentamer		Echovirus 18 missing two pentamers		Echovirus 18 missing three pentamers		Echo 30 missing one pentamer	
EMDB code	**EMD-0181**	**EMD-0182**	**EMD-0183**	**EMD-0184**	**EMD-0185**	**EMD-0186**	**EMD-0217**	**EMD-0187**	**EMD-0188**	**EMD-0189**	
PDB code	**6HBG**	**6HBH**	**6HBJ**	**-**	**6HBK**	**-**	**6HHT**	**-**	**6HBL**	**-**	
**Data collection and processing**											**Data collection and processing**
Magnification	75,000x	75,000x	75,000x	75,000x	75,000x	75,000x	75,000x	75,000x	75,000x	75,000x	Magnification
Voltage (kV)	300	300	300	300	300	300	300	300	300	300	Voltage (kV)
Electron exposure (e–/Å^2)	23.4	23.4	23.4	22.6	22.6	22.6	22.6	22.6	22.6	22.6	Electron exposure (e–/Å^2)
Defocus range (μm)	1—3	1—3	1—3	1—3	1—3	1—3	1—3	1—3	1—3	1—3	Defocus range (μm)
Pixel size (Å)	1.061	1.061	1.061	1.061	1.063	1.061	1.061	1.061	1.061	1.063	Pixel size (Å)
**Symmetry imposed**	**icosahedral**	**icosahedral**	**icosahedral**	**asymetric**	**fivefold**	**asymetric**	**twofold**	**asymetric**	**threefold**	**fivefold**	**Symmetry imposed**
Init. particle images (no.)	42,863	33,488	17,959	509,565	509,565	509,565	509,565	509,565	509,565	8.490	Init. particle images (no.)
Final particle images (no.)	10,062	31,197	8,511	3,563	3,563	7,635	7,635	13,635	13,635	235	Final particle images (no.)
**Map resolution (Å)**	**3.16**	**3.36**	**3.16**	**8.75**	**3.80**	**6.62**	**4.05**	**4.85**	**3.70**	**19.44**	**Map resolution (Å)**
FSC threshold	0.143	0.143	0.143	0.143	0.143	0.143	0.143	0.143	0.143	0.143	FSC threshold
Map sharp. *B* factor (Å^2)	-82.8	-163.98	-117.89	-233.50	-131.32	-114.57	-94.73	-116.77	-92.45	-	Map sharp. *B* factor (Å^2)
**Refinement**											**Refinement**
Initial model used	Echovirus 7	Echovirus 18	Echovirus 18	-	Echovirus 18	-	Echovirus 18	-	Echovirus 18	-	Initial model used
(PDB code)	(2X5I)	(6HBG)	(6HBH)		(6HBH)		(6HBH)		(6HBH)		(PDB code)
Initial model resolution (Å)	3.1	3.16	3.36	-	3.36	-	3.36	-	3.36	-	Initial model resolution (Å)
Model resol. range (Å)	∞ - 3.16	∞ - 3.36	∞ - 3.16	-	∞ - 3.80	-	∞ - 4.05	-	∞ - 3.70	-	Model resol. range (Å)
**Model composition**										-	**Model composition**
Non-hydrogen atoms	367 260	311 760	300 960	-	285 780	-	259 800	-	233 820	-	Non-hydrogen atoms
Protein residues	46 200	39 840	38 460	-	36 520	-	33 200	-	29 880	-	Protein residues
Ligands	120	-	-	-	-	-	-	-	-	-	Ligands
***B* factors (Å^2)**										-	***B* factors (Å^2)**
Protein	41.17	49.41	40.38	-	65.74	-	24.99	-	46.12	-	Protein
Ligand	42.7	-	-	-	-	-	-	-	-	-	Ligand
**R.m.s. deviations**										-	**R.m.s. deviations**
Bond lengths (Å)	0.004	0.003	0.003	-	0.003	-	0.003	-	0.001	-	Bond lengths (Å)
Bond angles (°)	0.736	0.688	0.636	-	0.725	-	0.697	-	0.697	-	Bond angles (°)
**Validation**										-	**Validation**
MolProbity score (percentile)	2.00 (100)	2.10 (100)	1.84 (100)	-	2.00 (100)	-	1.92 (100)	-	1.95 (100)	-	MolProbity score (percentile)
Clashscore (percentile)	14.31 (95)	10.91 (97)	7.91 (95)	-	7.83 (95)	-	6.99 (97)	-	7.59 (94)	-	Clashscore (percentile)
Poor rotamers (%)	0.3	0.0	0.0	-	0.0	-	0.0	-	0.0	-	Poor rotamers (%)
**Ramachandran plot**										-	**Ramachandran plot**
Favored (%)	95.14	90.21	93.76	-	89.27	-	90.44	-	90.45	-	Favored (%)
Allowed (%)	4.21	9.34	6.24	-	9.67	-	9.11	-	9.10	-	Allowed (%)
Disallowed (%)	0.65	0.45	0.00	-	1.06	-	0.45	-	0.45	-	Disallowed (%)
**R factors**											**R factors**
R work	0.323	0.318	0.332	-	0.376	-	0.451	-	0.465	-	R work
R free	0.325	0.323	0.336	-	0.374	-	0.454	-	0.467	-	R free

Supplementary table 2. Buried surface areas of inter-pentamer interfaces of native virions and activated particles of selected enteroviruses.

Virus	Burried surface area of pentamer in capsid (Å2)			
	Native virion		Activated particle	
	PBD		PDB	
Echovirus E18	6hbg	10 019	6hbh	6 599
Poliovirus type 1 (strain Mahoney)	1asj	11 108		
Human rhinovirus 16	1aym	10 591		
Bovine enterovirus (strain VG-5-27)	1bev	10 765		
Coxsackievirus B3 (strain Woodruff)	1cov	11 082		
Coxsackievirus A9 (strain Griggs)	1d4m	11 654		
Poliovirus type 2 (strain Lansing)	1eah	10 436		
Echovirus 1 (strain Farouk 1951)	1ev1	11 586		
Human rhinovirus 2	1fpn	10 384	3tn9	5 957
Echovirus 11	1h8t	11 218		
Swine vesicular disease virus	1mqt	10 462		
Human rhinovirus 16	1nd2	10 669		
Poliovirus type 3 (strain Leon)	1pvc	10 773		
Human rhinovirus 1A	1r1a	10 639		
Human rhinovirus 3	1rhi	10 563		
Coxsackievirus A21 (strain Coe)	1z7s	10 790		
Echovirus 7	2x5i	10 890		
Human enterovirus 71	3vbs	10 532	4n43	6 491
Coxsackievirus A24	4q4v	10 355		
Human rhinovirus 14	4rhv	10 470		
Enterovirus D68	4wm8	11 452	6aj2	7 163
Coxsackievirus A16	5c4w	11 186	4jgy	7 392
Rhinovirus C	5k0u	9 777		
Bovine enterovirus 2	5osn	10 829		
Coxsackievirus A10	6acu	11 318	6acy	5 012

Supplementary Table 3. List of flexible residues in native virion and activated particle of echovirus 18.

Type of particle	Chain	Flexible residues
Native virion	VP1	1-7, 77-82, 122-130, 277-287
	VP2	1-10
	VP3	
	VP4	1-31
Activated particle	VP1	1-46, 123-131, 277-287
	VP2	1-12, 27-30, 258-260
	VP3	74-77, 175-186, 231-239
