# Supplementary material for: Enterovirus particles expel capsid pentamers to enable genome release
Source: Nat Commun. 2019 Mar 8;10:1138. doi: 10.1038/s41467-019-09132-x (PMC6408523; doi:10.1038/s41467-019-09132-x)
Supplement: Supplementary file 3 — Description of Additional Supplementary Files [file 41467_2019_9132_MOESM3_ESM.pdf]

Description of Additional Supplementary Files

File Name: Supplementary Movie 1
Description: Molecular dynamics simulation of echovirus 18 genome release. Genome is shown in blue, outer capsid surface in orange, inner capsid surface in purple, beads at pentamer edges shown in dark and light gray, green and red represent attractive inter-pentamer interfaces.
